# 1,3-Bis(prop-2-yn-1-yl)-1*H*-anthra[1,2-*d*]imidazole-2,6,11(3*H*)-trione

**DOI:** 10.1107/S1600536813013688

**Published:** 2013-05-22

**Authors:** Zahra Afrakssou, Amal Haoudi, Frédéric Capet, Ahmed Mazzah, Christian Rolando, Lahcen El Ammari

**Affiliations:** a Laboratoire de Chimie Organique Appliquée, Université Sidi Mohamed, Ben Abdallah, Faculté des Sciences et Techniques, Route d’Immouzzer, BP 2202 Fès, Morocco; b Unité de Catalyse et de Chimie du Solide (UCCS), UMR 8181 Ecole Nationale Supérieure de Chimie de Lille, France; c USR 3290 Miniaturisation pour l’Analyse, la Synthèse et la Protéomique, 59655 Villeneuve d’Ascq Cedex, Université Lille 1, France; d Laboratoire de Chimie du Solide Appliquée, Faculté des Sciences, Université Mohammed V-Agdal, Avenue Ibn Battouta, BP 1014, Rabat, Morocco

## Abstract

In the title compound, C_21_H_12_N_2_O_3_, the fused-ring system is roughly planar, the largest deviation from the mean plane being 0.084 (2) Å. The two prop-2-yn-1-yl groups are almost perpendicular to the fused ring plane, making C—C—N—C torsion angles of −103.4 (2) and −105.3 (2)°, and point in opposite directions with respect to the plane. In the crystal, mol­ecules are linked by weak C—H⋯O hydrogen bonds, forming a three-dimensional network.

## Related literature

For background to the pharmacological activity and potential applications of anthra­quinones, see: Alves *et al.* (2004 ▶); Ellis *et al.* (2003 ▶); Boseggia *et al.* (2004 ▶); Mariappan & Basa (2011 ▶); Kadarkaraisamy *et al.* (2008 ▶). For similar compounds, see: Afrakssou *et al.* (2010 ▶, 2011 ▶); Guimarães *et al.* (2009 ▶).
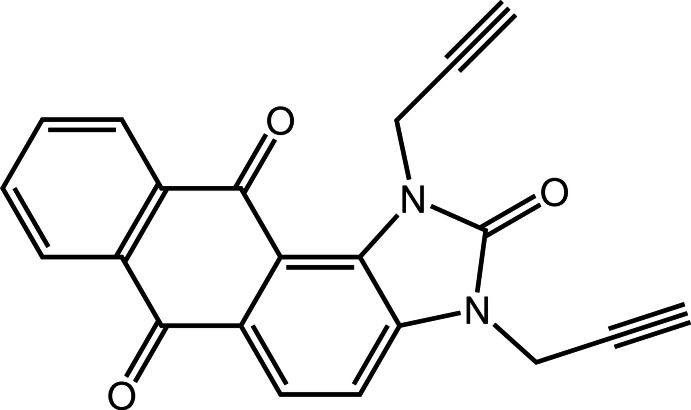


## Experimental

### 

#### Crystal data

C_21_H_12_N_2_O_3_
*M* *_r_* = 340.33
Monoclinic, 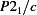

*a* = 16.6972 (5) Å
*b* = 4.5602 (1) Å
*c* = 21.2500 (5) Å
β = 96.352 (2)°
*V* = 1608.10 (7) Å^3^
*Z* = 4
Mo *K*α radiation
μ = 0.10 mm^−1^
*T* = 296 K
0.46 × 0.14 × 0.05 mm

#### Data collection

Bruker APEXII CCD diffractometer
20426 measured reflections
3177 independent reflections
2301 reflections with *I* > 2σ(*I*)
*R*_int_ = 0.033

#### Refinement

*R*[*F* ^2^ > 2σ(*F* ^2^)] = 0.044
*wR*(*F* ^2^) = 0.129
*S* = 1.02
3177 reflections
235 parameters
H-atom parameters constrained
Δρ_max_ = 0.33 e Å^−3^
Δρ_min_ = −0.25 e Å^−3^

### 

Data collection: *APEX2* (Bruker, 2009 ▶); cell refinement: *SAINT-Plus* (Bruker, 2009 ▶); data reduction: *SAINT-Plus*; program(s) used to solve structure: *SHELXS97* (Sheldrick, 2008 ▶); program(s) used to refine structure: *SHELXL97* (Sheldrick, 2008 ▶); molecular graphics: *ORTEP-3 for Windows* (Farrugia, 2012 ▶); software used to prepare material for publication: *PLATON* (Spek, 2009 ▶) and *publCIF* (Westrip, 2010 ▶).

## Supplementary Material

Crystal structure: contains datablock(s) I, global. DOI: 10.1107/S1600536813013688/im2432sup1.cif

Structure factors: contains datablock(s) I. DOI: 10.1107/S1600536813013688/im2432Isup2.hkl

Supplementary material file. DOI: 10.1107/S1600536813013688/im2432Isup3.cml

Additional supplementary materials:  crystallographic information; 3D view; checkCIF report

## Figures and Tables

**Table 1:** Hydrogen-bond geometry (Å, °)

*D*—H⋯*A*	*D*—H	H⋯*A*	*D*⋯*A*	*D*—H⋯*A*
C18—H18⋯O2^i^	0.93	2.31	3.165 (3)	152
C21—H21⋯O1^ii^	0.93	2.38	3.275 (3)	161
C2—H2⋯O3^iii^	0.93	2.62	3.333 (3)	134

Symmetry codes: (i) 
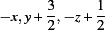
; (ii) 
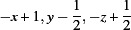
; (iii) 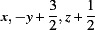.

**Fig. 1.**
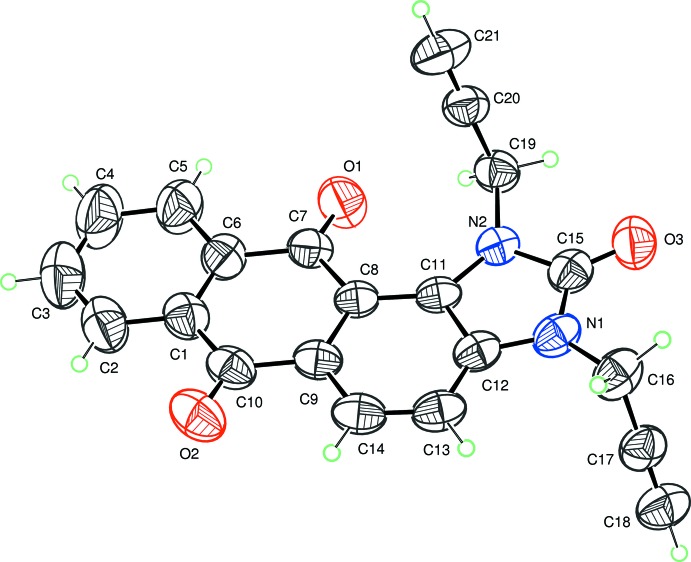
: Molecular structure of the title compound with the atom-labelling scheme. Displacement ellipsoids are drawn at the 50% probability level. H atoms are represented as small circles.

**Fig. 2.**
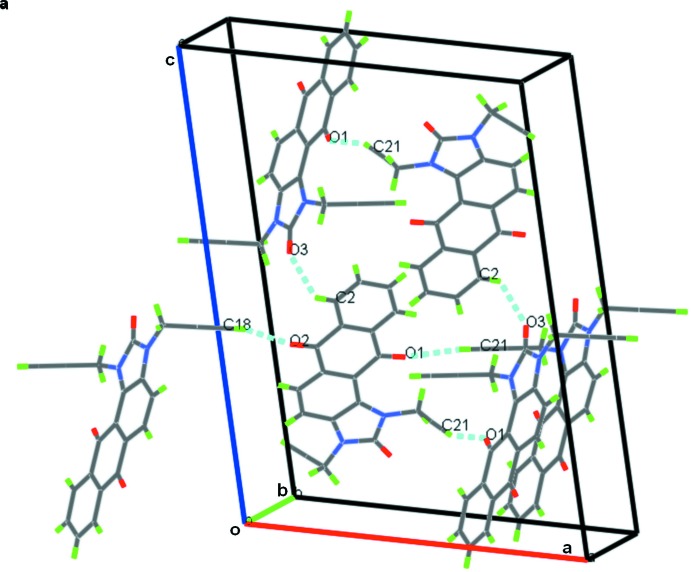
: Intermolecular interactions in the crystal structure of title compound. Hydrogen bonds are shown as dashed lines.

## supplementary crystallographic information

### Comment

Anthraquinone-containing extracts from different plant sources such as senna, cascara, aloe, frangula, and rhubarb have been found to have a wide variety of pharmacological activities such as antiinflammatory, wound healing, analgesic, antipyretic, antimicrobial, and antitumor activities (Alves *et al.*, 2004). Anthraquinone containing compounds are also important due to their potential applications as DNA intercalators (Ellis *et al.*, 2003; Boseggia *et al.*, 2004), as selective luminescent sensors of oxo-acids and metal ions when integrated into polyether chains (Mariappan & Basa, 2011) and as molecular switches (Kadarkaraisamy *et al.*, 2008).

So we are interested in the synthesis of new derivatives of anthra [1,2-*d*]imidazole-2,6, 11–trione and their biological activities (Afrakssou *et al.*, 2010; Afrakssou *et al.*, (2011); Guimarães *et al.*, 2009). The reactivity of propargyl bromide towards 1*H*-anthra [2, 1 - d] imidazole-2, 6, 11(3*H*)-trione under phase-transfer catalysis conditions using tetra *n*-butyl ammonium bromide (TBAB) as catalyst and potassium carbonate as base, leads to the formation of title compound in good yields (Scheme 1).

The four fused rings forming the molecule of the title compound are approximately planar, the largest deviation from the mean plane being 0.084 (2) Å at C10 (Fig. 1). The two prop-2-yn-1-yl (C16 to C18 and C19 to C21) groups are situated on opposite sides of the imidazole ring and are almost perpendicular to the fused rings plane, making C17–C16–N1–C15 and C20–C19–N2–C15 torsion angles of -103.4 (2) ° and -105.3 (2) °, respectively.

In the crystal, each molecule is linked to three adjacent molecules by intermolecular weak C18–H18···O2, C21–H21···O1 and C2–H2···O3 hydrogen bonds, forming a three-dimensional network (Fig. 2 and Table 2).

### Experimental

To a solution of 1*H*-anthra [2, 1 - d] imidazole-2, 6, 11(3*H*)-trione (0.05 g, 0.18 mmol), potassium carbonate (0.08 g, 0.56 mmol) and tetra n-butylammonium bromide (0.06 g, 0.018 mmol) in DMF (15 ml)) was added propargyl bromide (0.06 ml, 0.8 mmol). Stirring was continued at room temperature for 24 h. The mixture was filtered and the solvent removed. The residue was extracted with water. The organic compound was chromatographed on a column of silica gel with ethyl acetate-hexane (1/1) as eluent. Orange crystals were isolated when the solvent was allowed to evaporate (Yield: 65%).

### Refinement

All H atoms could be located in a difference Fourier map. However, they were placed in calculated positions with C—H = 0.93 Å (aromatic and methyne), and C—H = 0.97 Å (methylene) and refined as riding on their parent atoms with *U*_iso_(H) = 1.2 *U*_eq_ (C).

### Crystal data

**Table:**

C_21_H_12_N_2_O_3_	*F*(000) = 704
*M**_r_* = 340.33	*D*_x_ = 1.406 Mg m^−^^3^
Monoclinic, *P*2_1_/*c*	Melting point: 463 K
Hall symbol: -P 2ybc	Mo *K*α radiation, λ = 0.71073 Å
*a* = 16.6972 (5) Å	Cell parameters from 3177 reflections
*b* = 4.5602 (1) Å	θ = 2.5–26.0°
*c* = 21.2500 (5) Å	µ = 0.10 mm^−^^1^
β = 96.352 (2)°	*T* = 296 K
*V* = 1608.10 (7) Å^3^	Irregular shape, yellow
*Z* = 4	0.46 × 0.14 × 0.05 mm

### Data collection

**Table:**

Bruker APEXII CCD diffractometer	2301 reflections with *I* > 2σ(*I*)
Radiation source: fine-focus sealed tube	*R*_int_ = 0.033
Graphite monochromator	θ_max_ = 26.0°, θ_min_ = 2.4°
φ and ω scans	*h* = −20→19
20426 measured reflections	*k* = −5→5
3177 independent reflections	*l* = −26→25

### Refinement

**Table:**

Refinement on *F*^2^	Primary atom site location: structure-invariant direct methods
Least-squares matrix: full	Secondary atom site location: difference Fourier map
*R*[*F*^2^ > 2σ(*F*^2^)] = 0.044	Hydrogen site location: difference Fourier map
*wR*(*F*^2^) = 0.129	H-atom parameters constrained
*S* = 1.02	*w* = 1/[σ^2^(*F*_o_^2^) + (0.0555*P*)^2^ + 0.5235*P*] where *P* = (*F*_o_^2^ + 2*F*_c_^2^)/3
3177 reflections	(Δ/σ)_max_ < 0.001
235 parameters	Δρ_max_ = 0.33 e Å^−^^3^
0 restraints	Δρ_min_ = −0.25 e Å^−^^3^

### Special details

**Table:**

Geometry. All s.u.'s (except the s.u. in the dihedral angle between two l.s. planes) are estimated using the full covariance matrix. The cell s.u.'s are taken into account individually in the estimation of s.u.'s in distances, angles and torsion angles; correlations between s.u.'s in cell parameters are only used when they are defined by crystal symmetry. An approximate (isotropic) treatment of cell s.u.'s is used for estimating s.u.'s involving l.s. planes.
Refinement. Refinement of *F*^2^ against all reflections. The weighted *R*-factor *wR* and goodness of fit *S* are based on *F*^2^, conventional *R*-factors *R* are based on *F*, with *F* set to zero for negative *F*^2^. The threshold expression of *F*^2^ > σ(*F*^2^) is used only for calculating *R*-factors(gt) *etc*. and is not relevant to the choice of reflections for refinement. *R*-factors based on *F*^2^ are statistically about twice as large as those based on *F*, and *R*- factors based on all data will be even larger.

### Fractional atomic coordinates and isotropic or equivalent isotropic displacement parameters (Å^2^)

**Table:**

	*x*	*y*	*z*	*U*_iso_*/*U*_eq_	
C1	0.27182 (12)	0.6098 (4)	0.40077 (9)	0.0604 (5)	
C2	0.28450 (15)	0.4803 (6)	0.46051 (10)	0.0812 (7)	
H2	0.2480	0.3427	0.4725	0.097*	
C3	0.35059 (19)	0.5550 (7)	0.50163 (11)	0.0980 (9)	
H3	0.3581	0.4711	0.5417	0.118*	
C4	0.40550 (18)	0.7526 (7)	0.48372 (12)	0.1005 (9)	
H4	0.4507	0.7995	0.5115	0.121*	
C5	0.39427 (14)	0.8828 (6)	0.42474 (10)	0.0811 (7)	
H5	0.4320	1.0164	0.4130	0.097*	
C6	0.32708 (12)	0.8154 (4)	0.38289 (8)	0.0581 (5)	
C7	0.31375 (10)	0.9681 (4)	0.32094 (8)	0.0521 (5)	
C8	0.24435 (9)	0.8852 (4)	0.27529 (8)	0.0447 (4)	
C9	0.18813 (10)	0.6776 (4)	0.29414 (8)	0.0505 (4)	
C10	0.20212 (12)	0.5247 (4)	0.35604 (9)	0.0595 (5)	
C11	0.22732 (9)	1.0111 (4)	0.21489 (8)	0.0444 (4)	
C12	0.15499 (10)	0.9328 (4)	0.17752 (8)	0.0500 (4)	
C13	0.09989 (11)	0.7381 (4)	0.19747 (10)	0.0596 (5)	
H13	0.0521	0.6955	0.1723	0.072*	
C14	0.11783 (11)	0.6094 (4)	0.25560 (10)	0.0603 (5)	
H14	0.0822	0.4735	0.2696	0.072*	
C15	0.21803 (11)	1.2631 (4)	0.12116 (9)	0.0551 (5)	
C16	0.09096 (12)	1.0549 (5)	0.06690 (9)	0.0677 (6)	
H16A	0.0776	0.8490	0.0610	0.081*	
H16B	0.1133	1.1226	0.0293	0.081*	
C17	0.01719 (12)	1.2201 (5)	0.07407 (9)	0.0592 (5)	
C18	−0.04129 (13)	1.3540 (5)	0.07967 (11)	0.0738 (6)	
H18	−0.0878	1.4604	0.0841	0.089*	
C19	0.34510 (10)	1.3565 (4)	0.18615 (9)	0.0546 (5)	
H19A	0.3465	1.5073	0.1541	0.066*	
H19B	0.3530	1.4511	0.2272	0.066*	
C20	0.41099 (11)	1.1519 (4)	0.18097 (9)	0.0565 (5)	
C21	0.46571 (13)	0.9943 (6)	0.17751 (11)	0.0821 (7)	
H21	0.5092	0.8691	0.1748	0.099*	
N1	0.15159 (9)	1.0865 (4)	0.12144 (7)	0.0558 (4)	
N2	0.26525 (8)	1.2155 (3)	0.17856 (7)	0.0482 (4)	
O1	0.35778 (9)	1.1731 (4)	0.31003 (6)	0.0812 (5)	
O2	0.15706 (10)	0.3264 (3)	0.36931 (8)	0.0825 (5)	
O3	0.23256 (9)	1.4307 (4)	0.07918 (6)	0.0718 (4)	

### Atomic displacement parameters (Å^2^)

**Table:**

	*U*^11^	*U*^22^	*U*^33^	*U*^12^	*U*^13^	*U*^23^
C1	0.0692 (12)	0.0584 (12)	0.0556 (11)	0.0081 (10)	0.0164 (9)	−0.0032 (9)
C2	0.1003 (18)	0.0830 (16)	0.0623 (13)	0.0040 (14)	0.0186 (13)	0.0081 (12)
C3	0.126 (2)	0.107 (2)	0.0583 (14)	0.0071 (19)	−0.0008 (15)	0.0138 (14)
C4	0.108 (2)	0.119 (2)	0.0669 (15)	−0.0052 (19)	−0.0214 (14)	0.0100 (16)
C5	0.0779 (15)	0.0963 (18)	0.0649 (13)	−0.0095 (13)	−0.0110 (11)	0.0053 (13)
C6	0.0610 (11)	0.0617 (12)	0.0516 (10)	0.0051 (10)	0.0058 (8)	−0.0054 (9)
C7	0.0453 (10)	0.0593 (11)	0.0525 (10)	−0.0035 (9)	0.0084 (8)	−0.0054 (9)
C8	0.0409 (9)	0.0431 (9)	0.0511 (9)	0.0031 (7)	0.0096 (7)	−0.0088 (8)
C9	0.0474 (10)	0.0460 (10)	0.0600 (10)	0.0002 (8)	0.0138 (8)	−0.0108 (9)
C10	0.0631 (12)	0.0512 (11)	0.0681 (12)	0.0017 (9)	0.0243 (10)	−0.0045 (10)
C11	0.0377 (8)	0.0418 (9)	0.0541 (9)	0.0030 (7)	0.0073 (7)	−0.0110 (8)
C12	0.0442 (9)	0.0505 (10)	0.0548 (10)	0.0048 (8)	0.0028 (7)	−0.0144 (9)
C13	0.0439 (10)	0.0633 (12)	0.0702 (12)	−0.0056 (9)	0.0003 (8)	−0.0207 (10)
C14	0.0505 (10)	0.0568 (11)	0.0754 (13)	−0.0096 (9)	0.0145 (9)	−0.0127 (10)
C15	0.0545 (11)	0.0581 (11)	0.0528 (10)	0.0102 (9)	0.0059 (8)	−0.0075 (10)
C16	0.0679 (13)	0.0758 (14)	0.0558 (11)	0.0085 (11)	−0.0094 (9)	−0.0183 (10)
C17	0.0515 (11)	0.0669 (13)	0.0565 (10)	−0.0073 (10)	−0.0066 (8)	−0.0058 (10)
C18	0.0527 (12)	0.0854 (16)	0.0807 (14)	−0.0005 (12)	−0.0038 (10)	−0.0060 (13)
C19	0.0516 (10)	0.0524 (11)	0.0604 (10)	−0.0036 (8)	0.0082 (8)	0.0000 (9)
C20	0.0461 (10)	0.0659 (12)	0.0573 (10)	−0.0024 (9)	0.0049 (8)	−0.0042 (10)
C21	0.0526 (12)	0.1004 (18)	0.0918 (16)	0.0151 (13)	0.0003 (11)	−0.0172 (14)
N1	0.0493 (9)	0.0611 (10)	0.0550 (9)	0.0049 (7)	−0.0038 (7)	−0.0128 (8)
N2	0.0423 (8)	0.0501 (8)	0.0519 (8)	0.0013 (6)	0.0036 (6)	−0.0032 (7)
O1	0.0712 (9)	0.1062 (13)	0.0632 (8)	−0.0379 (9)	−0.0060 (7)	0.0079 (8)
O2	0.0878 (11)	0.0711 (10)	0.0930 (11)	−0.0163 (9)	0.0289 (9)	0.0080 (9)
O3	0.0808 (10)	0.0764 (10)	0.0579 (8)	0.0050 (8)	0.0058 (7)	0.0079 (8)

### Geometric parameters (Å, º)

**Table:**

C1—C2	1.395 (3)	C12—N1	1.378 (2)
C1—C6	1.397 (3)	C12—C13	1.379 (3)
C1—C10	1.471 (3)	C13—C14	1.370 (3)
C2—C3	1.373 (3)	C13—H13	0.9300
C2—H2	0.9300	C14—H14	0.9300
C3—C4	1.369 (4)	C15—O3	1.219 (2)
C3—H3	0.9300	C15—N1	1.371 (2)
C4—C5	1.381 (3)	C15—N2	1.394 (2)
C4—H4	0.9300	C16—N1	1.459 (2)
C5—C6	1.387 (3)	C16—C17	1.466 (3)
C5—H5	0.9300	C16—H16A	0.9700
C6—C7	1.484 (3)	C16—H16B	0.9700
C7—O1	1.227 (2)	C17—C18	1.169 (3)
C7—C8	1.476 (2)	C18—H18	0.9300
C8—C11	1.406 (2)	C19—C20	1.456 (3)
C8—C9	1.421 (2)	C19—N2	1.473 (2)
C9—C14	1.390 (3)	C19—H19A	0.9700
C9—C10	1.484 (3)	C19—H19B	0.9700
C10—O2	1.229 (2)	C20—C21	1.171 (3)
C11—N2	1.405 (2)	C21—H21	0.9300
C11—C12	1.416 (2)		
			
C2—C1—C6	119.6 (2)	N1—C12—C11	107.92 (16)
C2—C1—C10	120.4 (2)	C13—C12—C11	123.15 (18)
C6—C1—C10	120.03 (18)	C14—C13—C12	117.79 (17)
C3—C2—C1	120.3 (2)	C14—C13—H13	121.1
C3—C2—H2	119.9	C12—C13—H13	121.1
C1—C2—H2	119.9	C13—C14—C9	121.41 (18)
C4—C3—C2	120.2 (2)	C13—C14—H14	119.3
C4—C3—H3	119.9	C9—C14—H14	119.3
C2—C3—H3	119.9	O3—C15—N1	126.71 (18)
C3—C4—C5	120.5 (2)	O3—C15—N2	126.79 (18)
C3—C4—H4	119.8	N1—C15—N2	106.50 (16)
C5—C4—H4	119.8	N1—C16—C17	112.56 (15)
C4—C5—C6	120.4 (2)	N1—C16—H16A	109.1
C4—C5—H5	119.8	C17—C16—H16A	109.1
C6—C5—H5	119.8	N1—C16—H16B	109.1
C5—C6—C1	119.07 (19)	C17—C16—H16B	109.1
C5—C6—C7	119.76 (19)	H16A—C16—H16B	107.8
C1—C6—C7	121.15 (17)	C18—C17—C16	179.4 (3)
O1—C7—C8	120.95 (17)	C17—C18—H18	180.0
O1—C7—C6	119.40 (17)	C20—C19—N2	113.23 (15)
C8—C7—C6	119.51 (16)	C20—C19—H19A	108.9
C11—C8—C9	117.12 (15)	N2—C19—H19A	108.9
C11—C8—C7	124.07 (15)	C20—C19—H19B	108.9
C9—C8—C7	118.71 (16)	N2—C19—H19B	108.9
C14—C9—C8	121.60 (18)	H19A—C19—H19B	107.7
C14—C9—C10	117.17 (17)	C21—C20—C19	177.8 (2)
C8—C9—C10	121.23 (16)	C20—C21—H21	180.0
O2—C10—C1	120.4 (2)	C15—N1—C12	110.27 (15)
O2—C10—C9	120.58 (19)	C15—N1—C16	123.02 (17)
C1—C10—C9	118.97 (17)	C12—N1—C16	126.52 (17)
N2—C11—C8	135.60 (15)	C15—N2—C11	109.78 (14)
N2—C11—C12	105.52 (15)	C15—N2—C19	116.43 (15)
C8—C11—C12	118.87 (16)	C11—N2—C19	133.40 (14)
N1—C12—C13	128.92 (16)		
			
C6—C1—C2—C3	0.3 (3)	C9—C8—C11—C12	2.0 (2)
C10—C1—C2—C3	178.5 (2)	C7—C8—C11—C12	−174.30 (15)
C1—C2—C3—C4	−1.4 (4)	N2—C11—C12—N1	0.18 (18)
C2—C3—C4—C5	1.2 (5)	C8—C11—C12—N1	179.18 (14)
C3—C4—C5—C6	0.1 (4)	N2—C11—C12—C13	−178.94 (15)
C4—C5—C6—C1	−1.3 (4)	C8—C11—C12—C13	0.1 (3)
C4—C5—C6—C7	177.0 (2)	N1—C12—C13—C14	179.16 (18)
C2—C1—C6—C5	1.0 (3)	C11—C12—C13—C14	−1.9 (3)
C10—C1—C6—C5	−177.15 (19)	C12—C13—C14—C9	1.7 (3)
C2—C1—C6—C7	−177.14 (18)	C8—C9—C14—C13	0.4 (3)
C10—C1—C6—C7	4.7 (3)	C10—C9—C14—C13	−179.65 (17)
C5—C6—C7—O1	−7.3 (3)	N1—C16—C17—C18	78 (22)
C1—C6—C7—O1	170.83 (18)	N2—C19—C20—C21	−169 (100)
C5—C6—C7—C8	176.98 (18)	O3—C15—N1—C12	−178.55 (18)
C1—C6—C7—C8	−4.8 (3)	N2—C15—N1—C12	0.87 (19)
O1—C7—C8—C11	5.9 (3)	O3—C15—N1—C16	6.1 (3)
C6—C7—C8—C11	−178.50 (16)	N2—C15—N1—C16	−174.45 (15)
O1—C7—C8—C9	−170.35 (17)	C13—C12—N1—C15	178.39 (17)
C6—C7—C8—C9	5.3 (2)	C11—C12—N1—C15	−0.67 (19)
C11—C8—C9—C14	−2.3 (2)	C13—C12—N1—C16	−6.5 (3)
C7—C8—C9—C14	174.22 (16)	C11—C12—N1—C16	174.46 (16)
C11—C8—C9—C10	177.82 (15)	C17—C16—N1—C15	−103.4 (2)
C7—C8—C9—C10	−5.7 (2)	C17—C16—N1—C12	82.1 (2)
C2—C1—C10—O2	−4.4 (3)	O3—C15—N2—C11	178.67 (17)
C6—C1—C10—O2	173.79 (18)	N1—C15—N2—C11	−0.75 (19)
C2—C1—C10—C9	176.93 (18)	O3—C15—N2—C19	−7.5 (3)
C6—C1—C10—C9	−4.9 (3)	N1—C15—N2—C19	173.08 (14)
C14—C9—C10—O2	6.9 (3)	C8—C11—N2—C15	−178.39 (18)
C8—C9—C10—O2	−173.18 (17)	C12—C11—N2—C15	0.35 (18)
C14—C9—C10—C1	−174.42 (16)	C8—C11—N2—C19	9.2 (3)
C8—C9—C10—C1	5.5 (3)	C12—C11—N2—C19	−172.03 (16)
C9—C8—C11—N2	−179.39 (17)	C20—C19—N2—C15	−105.30 (18)
C7—C8—C11—N2	4.3 (3)	C20—C19—N2—C11	66.7 (2)

### Hydrogen-bond geometry (Å, º)

**Table:**

*D*—H···*A*	*D*—H	H···*A*	*D*···*A*	*D*—H···*A*
C18—H18···O2^i^	0.93	2.31	3.165 (3)	152
C21—H21···O1^ii^	0.93	2.38	3.275 (3)	161
C2—H2···O3^iii^	0.93	2.62	3.333 (3)	134

Symmetry codes: (i) −*x*, *y*+3/2, −*z*+1/2; (ii) −*x*+1, *y*−1/2, −*z*+1/2; (iii) *x*, −*y*+3/2, *z*+1/2.
